# Supplementary material for: A Multifunctional Coating on Sulfur-Containing Carbon-Based Anode for High-Performance Sodium-Ion Batteries
Source: Molecules. 2023 Apr 10;28(8):3335. doi: 10.3390/molecules28083335 (PMC10142203; doi:10.3390/molecules28083335)
Supplement: Supplementary file 1 [file molecules-28-03335-s001.zip › molecules-2275465-supplementary-Revise.pdf]

A Multifunctional Coating on Sulfur-containing Carbon-based Anode for High-performance Sodium-ion Batteries

Lin Zhu [1,2], Yin Bo [2,] * Yuting Zhang [2], Qian Wu [2], Hongqiang Xu [2], Haojie Duan [2,] Meiqin Shi [1], *and Haiyong He [2,] *

[1] School of Chemical Engineering Zhejiang University of Technology, Hangzhou, 310014, P. R. China; zhulin@nimte.ac.cn

[2] Ningbo Institute of Materials Technology and Engineering, Chinese Academy of Sciences, Ningbo 315201, P. R. China; zhangyuting@nimte.ac.cn (Y.Z.); wuqian20@nimte.ac.cn (Q.W.); xuhongqiang@nimte.ac.cn (H.X.); duanhaojie@nimte.ac.cn (H.D.); hehai-yong@nimte.ac.cn (H.H.)

* Correspondence: yinbo@nimte.ac.cn (B.Y.); smq@zjut.edu.cn (M.Q.); hehaiyong@nimte.ac.cn (H.H.)

Table S1. Summary of some different carbon-based materials for SIBs

Number	Material	Reversible capacity (mAh g^{-1}, at A g^{-1})	ICE (%)	Reference
1	G-NCs	329 (0.05 A g^{-1})	32	Ref. [51]
2	Natural graphite	127 (0.1 A g^{-1})	-	Ref.[52]
3	A-CG	280 (0.04 A g^{-1})	38	Ref. [53]
4	HPCCNFs-1	215 (0.1 A g^{-1})	25.3	Ref.[15]
5	P-CNSs	237.5 (0.05 A g^{-1})	42.65	Ref.[37]
6	HCS-1900	295 (0.05 A g^{-1})	68.8	Ref. [54]
7	SNMHCSs	240 (0.05 A g^{-1})	29	Ref. [55]
8	PC-800	263.2 (0.05 A g^{-1})	79.6	Ref. [56]

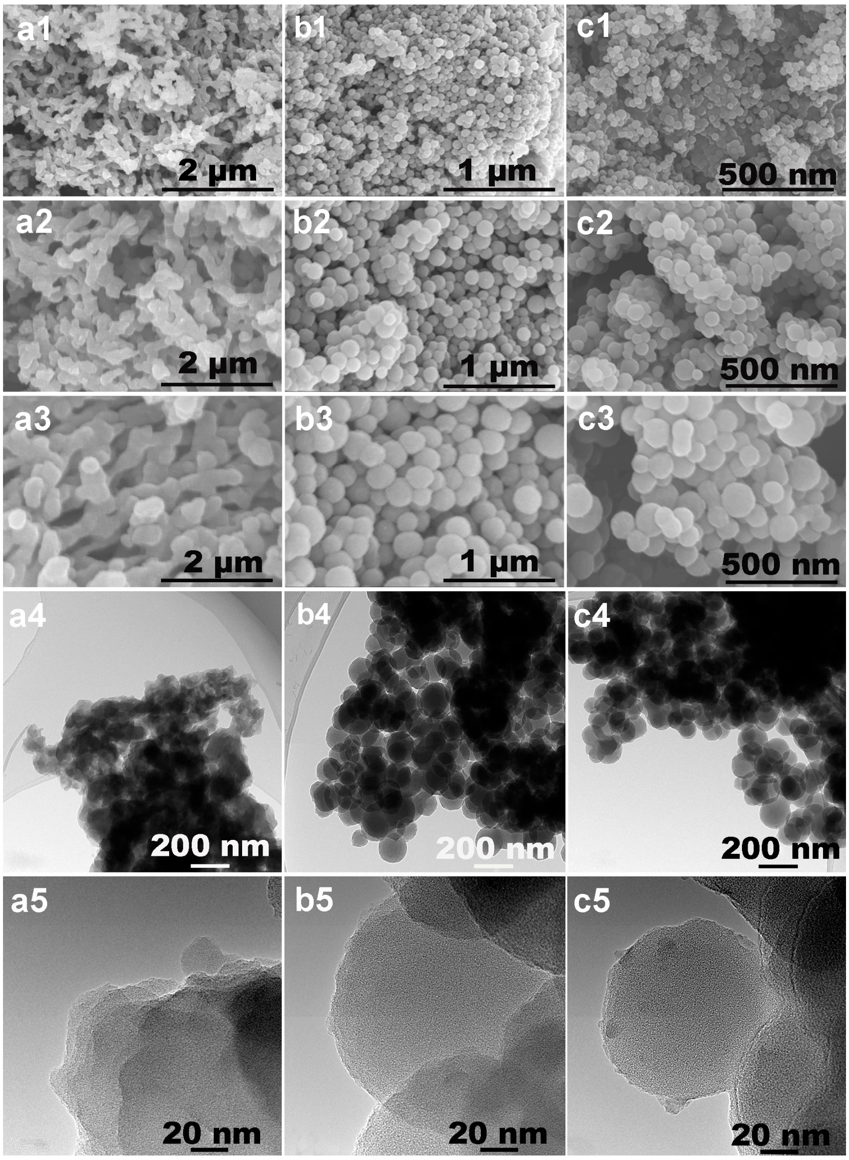

Figure S1. The SEM images of **a1, a2, a3**) NSC, **b1, b2, b3**) SGCS and **c1, c2, c3**) GCS at different magnifications. TEM and HRTEM images of **a4, a5**) NSC, **b4, b5**) SGCS and **c4, c5**) GCS.

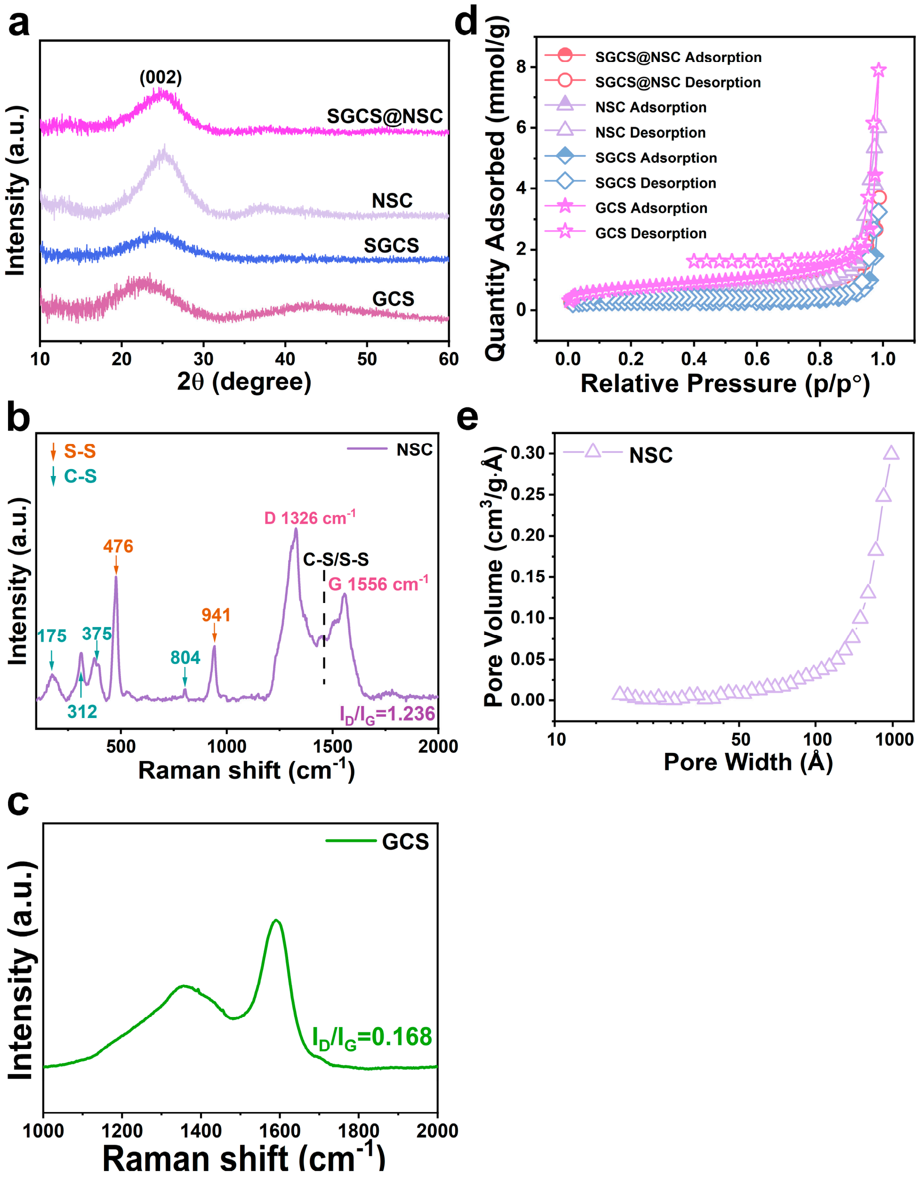

Figure S2. (**a**) X-ray diffraction patterns of SGCS@NSC, NSC, SGCS, and GCS. (**b, c**) Raman scattering spectra of NSC and GCS. (**d**) N2 adsorption-desorption isotherms of SGCS@NSC, NSC, SGCS, and GCS. (**e**) Pore distribution curves of NSC.

Table S2. Analysis of sulfur bonding area ratio of SGCS@NSC and SGCS.

Sulfur bonding	SGCS@NSC	SGCS
**HS_xC-**	8.1	
**C-S**	70.3	54.5
**S-S**	11.3	29.1
**Sulfate**	10.3	16.3

Table S3. Analysis of carbon bonding area ratio of SGCS@NSC and SGCS.

Carbon bonding	SGCS@NSC	SGCS
**C-C**	35.9	36.3
**C-N/C-S**	18.0	12.5
**C=O/C=N**	46.0	51.2

Table S4. Specific surface areas and pore volume analysis of SGCS@NSC, SGCS, GCS, and NSC.

Samples	S_{BET}(m^2/g)	V_{pore}(cm^3/g)	R_{pore}(nm）
**SGCS@NSC**	34.956	0.128	3.683
**SGCS**	18.670	0.113	6.052
**GCS**	56.147	0.274	4.880
**NSC**	31.168	0.208	8.984

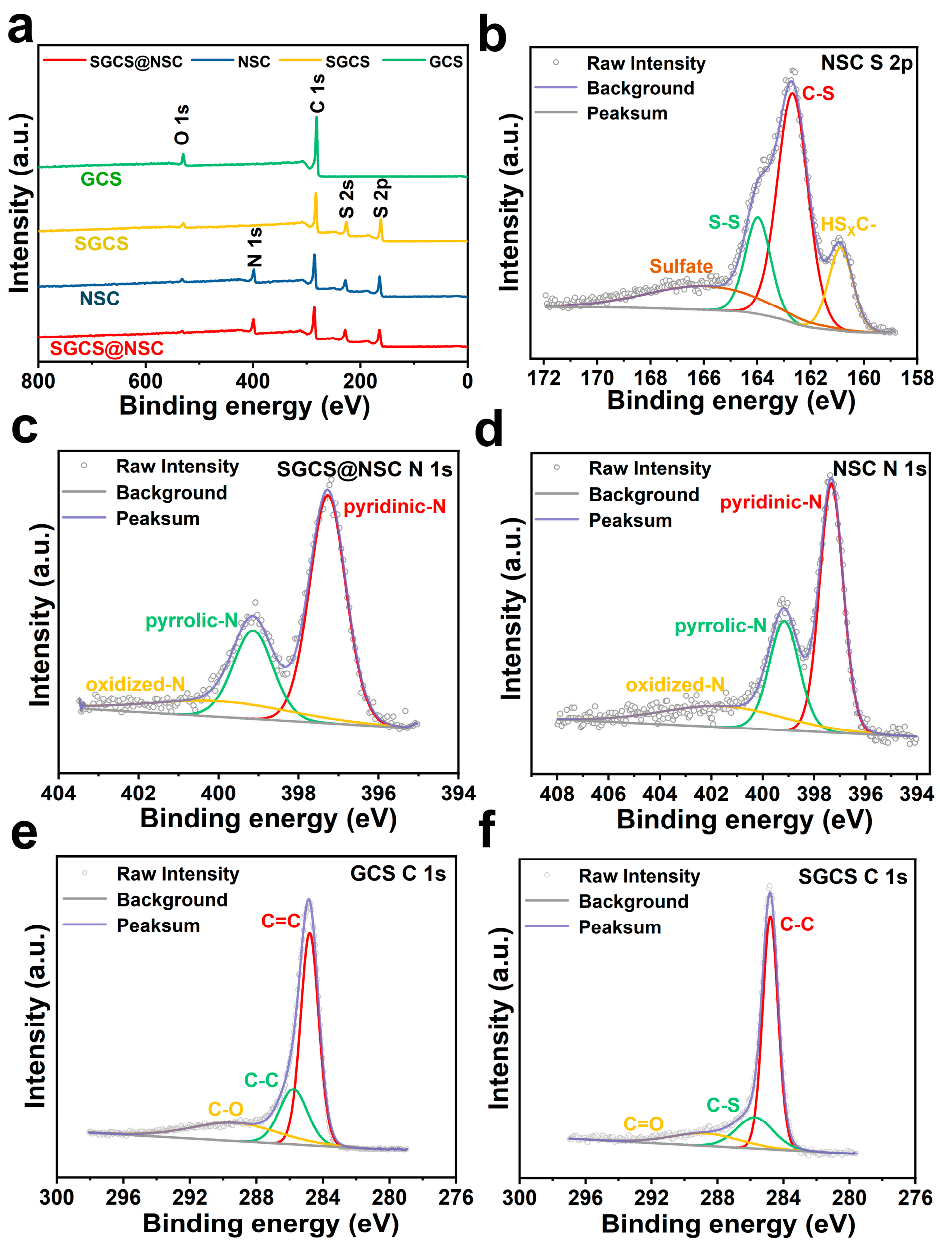

Figure S3. (**a**) Typical XPS survey spectra. (**b, d, f**) The high-resolution XPS of S 2p, N 1s, and C 1s for NSC, respectively. (**c**) The high-resolution XPS of N 1s for SGCS@NSC. (**e**) The high-resolution XPS of C 1s for GCS

Table S5. Element composition and ratio of the surface of four groups of samples calculated from XPS data (wt.%).

Samples	C（at%）	N（at%）	S（at%）	O（at%）
**SGCS@NSC**	63.019	12.637	19.075	2.471
**NSC**	61.019	16.542	19.860	1.813
**SGCS**	71.471		22.083	5.446
**GCS**	92.808			7.192

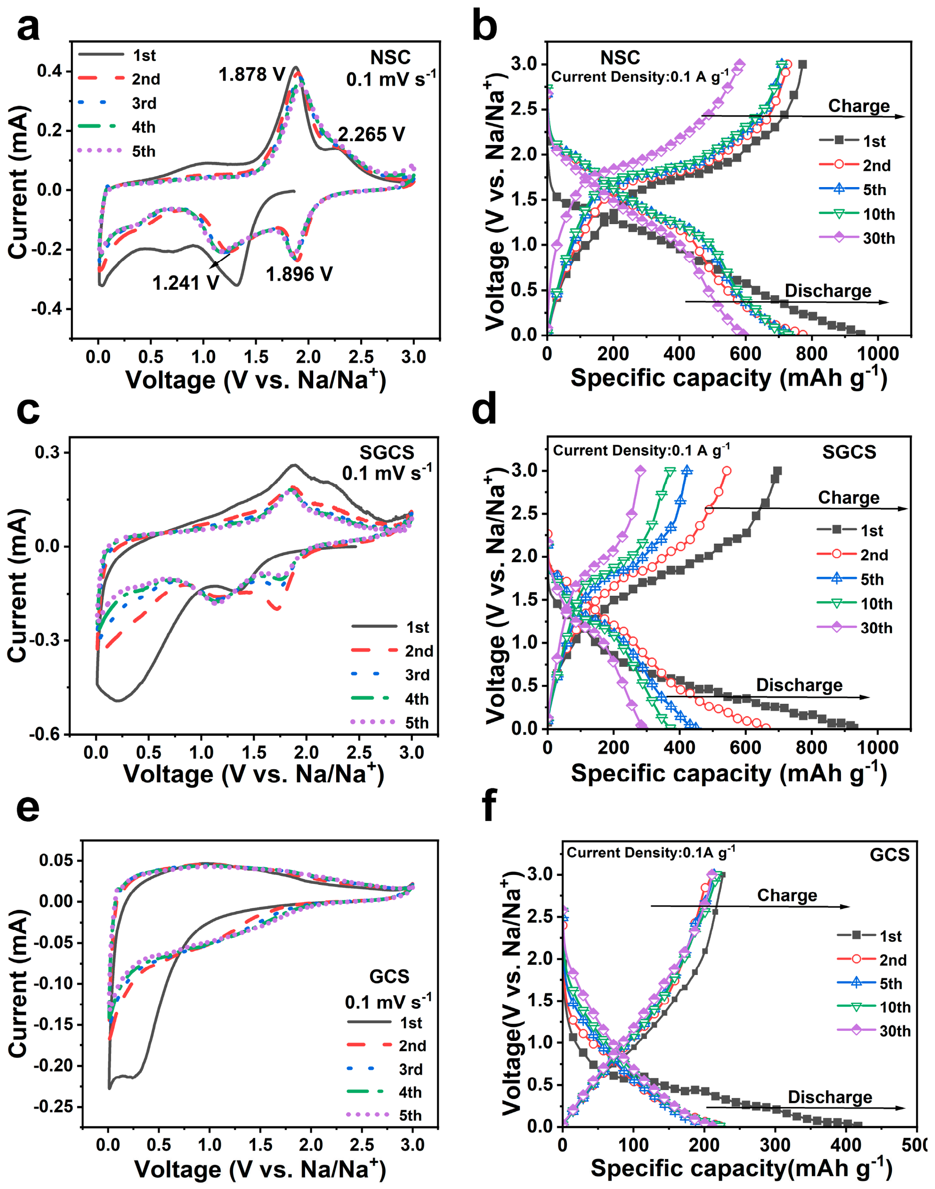

Figure S4. The first five successive CV curves of **a**) NSC, **c**) SGCS, and **e**) GCS at the scan rate of 0.1 mV s^{-1}. Galvanostatic discharge-charge profiles (100 mA g^{-1}) at the 1st, 2nd, 5th, 10th, and 30th cycles of **b**) NSC, **d**) SGCS, and **f**) GCS.

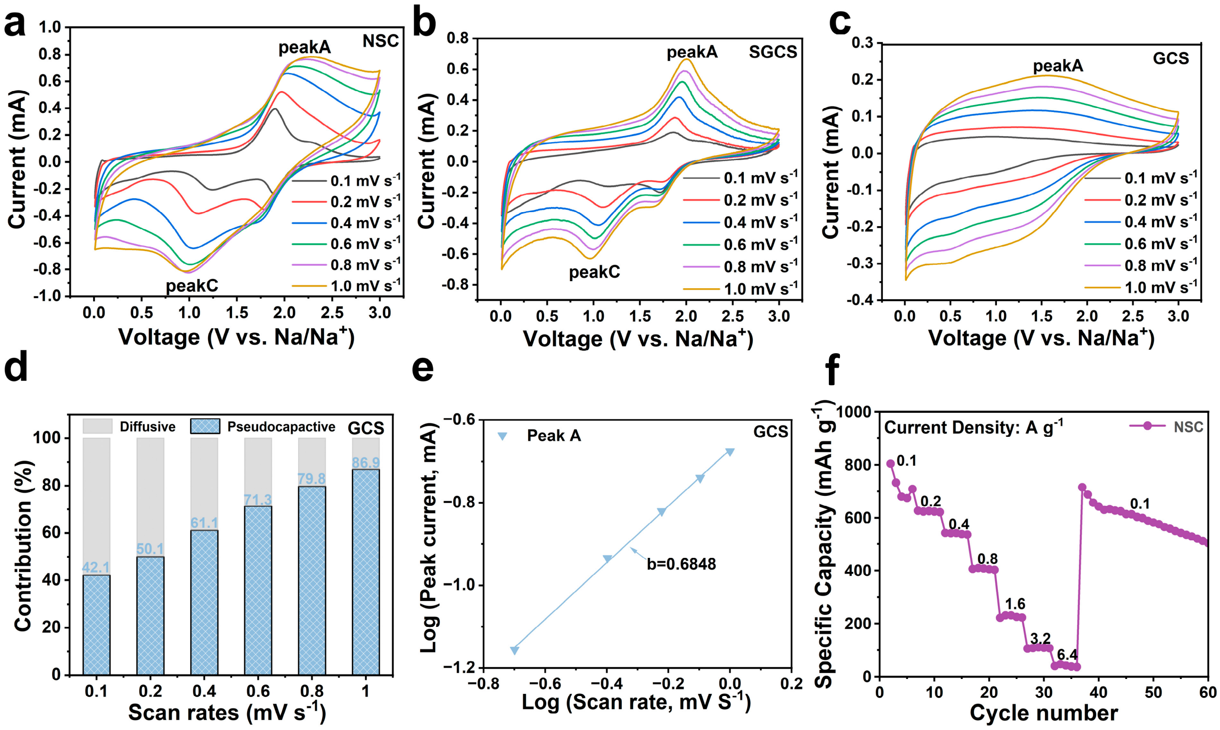

Figure S5. CV curves at different scan rates of **a**) NSC, **b**) SGCS, and **c**) GCS from 0.1 to 1.0 mV s^{-1}. (**d**) Normalized pseudocapacitive contribution rations of GCS at scan rates from 0.1 to 1.0 mV s^{-1}. (**e**) Log (i) versus log (v) plots of GCS at selected peak currents. (**f**) Rate capability of NSC electrodes at increasing current densities from 0.1 to 6.4 A g^{-1}.

The galvanostatic intermittent titration technique is used to calculate the Na^+ diffusion coefficient according to the following formula (S1):

$$D = \left(\frac{\Delta E_s}{\Delta E_t}\right)\frac{4L^2}{\pi\tau} \qquad \text{(S1)}$$

In the formula, ΔE_srepresents the change in steady-state potential of a current pulse (V), $\Delta E_t$is the elimination of constant current pulse potential (V) changes after iR drops, t is the duration of the current pulse, τ is the relaxation time, and L is the diffusion length of Na^+[42,57].

Table S6. The EIS fitting parameters of SGCS@NSC, NSC, SGCS, and GCS.

Samples	$R_s(\Omega)$	$R_{ct}(\Omega)$
**SGCS@NSC**	4.9	637.2
**NSC**	7.832	975.1
**SGCS**	11	1875
**GCS**	13.1	981
